# Modulation of Aryl Hydrocarbon Receptor Expression Alleviated Neuropathic Pain in a Chronic Constriction Nerve Injury Animal Model

**DOI:** 10.3390/ijms231911255

**Published:** 2022-09-24

**Authors:** Meei-Ling Sheu, Liang-Yi Pan, Jason Sheehan, Meng-Yin Yang, Hung-Chuan Pan

**Affiliations:** 1 Institute of Biomedical Sciences, National Chung-Hsing University, Taichung 40227, Taiwan; 2 Faculty of Medicine, Kaohsiung Medical University, Kaohsiung 80708, Taiwan; 3 Department of Neurosurgery, University of Virginia, Charlottesville, VA 22903, USA; 4 Department of Neurosurgery, Taichung Veterans General Hospital, Taichung 40201, Taiwan; 5 Department of Medical Research, Taichung Veterans General Hospital, Taichung 40201, Taiwan

**Keywords:** aryl hydrocarbon receptor, neuropathic pain, chronic constriction injury

## Abstract

Neuropathic pain is well known to occur after damage to the somatosensory system. Aryl hydrocarbon receptor (AhR) has neuroprotective effects when the central nervous system is subjected to internal and external stimulations. However, the exact mechanism by which AhR regulates neuropathic pain is poorly understood. Nerve explant culture and the chronic constrictive nerve injury (CCI) model in wild or AhR-knockout mice were used in this study. In the nerve explant culture, the ovoid number increased in the AhR−/− condition and was decreased by omeprazole (AhR agonist) in a dose-dependent manner. Increased nerve degeneration and the associated inflammation response appeared in the AhR−/− condition, and these changes were attenuated by omeprazole. High expression of AhR in the injured nerve was noted after CCI. Deletion of AhR aggravated nerve damages and this was restored by omeprazole. Deletion of AhR increased NGF expression and reduced axon number in the paw skin, but this was attenuated by omeprazole. A highly expressed inflammation reaction over the dorsal spinal cord, somatosensory cortex, and hippocampus was noted in the AhR-deleted animals. Administration of omeprazole attenuated not only the inflammatory response, but also the amplitude of somatosensory evoked potential. Deletion of AhR further aggravated the neurobehavior compared with the wild type, but such behavior was attenuated by omeprazole. Chronic constrictive nerve injury augmented AhR expression of the injured nerve, and AhR deletion worsened the damage, while AhR agonist omeprazole counteracted such changes. AhR agonists could be potential candidates for neuropathic pain treatment.

## 1. Introduction

Neuropathic pain is a sustained and nonreversible condition, typically a direct after-effect of a lesion or disease of the somatosensory system. By definition, it is the pain initiated or caused by a primary lesion or dysfunction of the nervous system [1,2]. Epidemic surveys show that 8% of the general population experience neuropathic pain, which may markedly affect their lives psychologically, physically, and socially. This extraordinary pain typically responds poorly to analgesics like anti-inflammatory and opiate drugs [1]. Therefore, neuropathic pain remains quite a substantial challenge for scientists and clinicians. In general, neuropathic pain is difficult to treat and responds poorly to current therapies. Due to its complexity, neuropathic pain does not respond to traditional analgesics, such as anti-inflammatory antagonists and opiates. Today, no completely effective treatment is available for neuropathic pain [3,4].

Aryl hydrocarbon receptor (AhR) is a protein in the PAS (Per-ARNT-Sim) family, with a basic helix–loop–helix domain. AhR is present in the cytoplasm of many vertebrate cells as part of a complex. The complex contains a dimer of the chaperone heat shock protein (HSP) 90, an immunophilin-like protein called X-associated protein (XAP) 2 or AIP1, the phosphoprotein p23, and the non-receptor protein tyrosine kinase (also known as pp60src) [5]. AhR regulates neural functions, implicated mainly by effects of xenobiotics and through the AhR ligand, 2,3,7,8-tetrachlorodibenzo-p-dioxin (TCDD), which affects neuronal proliferation, differentiation, and survival [6]. AhR expression is characterized in animal models based on evidence from immunohistochemistry and in situ hybridization [7]. Specific temporal and spatial patterns of the AhR messenger RNA (mRNA) are expressed in brain regions of the mouse-like cortex, hippocampus, cerebellum, olfactory bulb; the rostral migratory stream, and brain stem; and in the hypothalamus/pituitary axis [8,9]. Astrocytes and endothelial cells isolated from the murine blood–brain barrier also express the AhR protein, indicating that the expression is not restricted to neuronal progenitors or neurons [10,11]. AhR is also expressed in glial cells [12], and is integrated itself in neuronal survival [13]. AhR expressions in the brain also exhibit chrono-periodicity and periodic variations in the level of the AhR mRNA at the murine suprachiasmatic nucleus [14,15]. Regulation of AhR expression in the brain depends not only on internal stimuli, but also on external stimulation such as traumatic brain injury (TBI) or strokes [16,17,18]. 

The initiation and maintenance of neuropathic pain involve not only intrinsic neuronal pathways but also the peripheral immune system [19]. Upon nerve injury, inflammatory responses occur from the skin to the spinal cord and even the somatosensory cortex [20,21,22]. External stimuli caused by chronic constrictive injury likely contribute to widely distributed AhR expressions from the skin to the brain, leading to neuropathic pain. Thus far, few studies have been published concerning the role of AhR in regulating neuropathic pain. 

In search of a pharmaceutical strategy to reduce neuropathic pain, we investigate putative AhR-activating pharmaceuticals. One class of drugs having pleiotropic effects linked to the AhR pathway is the proton pump inhibitors (PPIs). PPIs are widely used in treating gastroesophageal reflux disease (GERD) and peptic ulcers [23]. Omeprazole (Prilosec) is a benzimidazole derivative and an AhR activator and ligand [24,25,26]. In this study, we investigated specifically the AhR agonist omeprazole in reducing neuropathic pain through activation of AhR expression.

Here, we continue to use our reported animal model of chronic constriction nerve injury [20,22,23]. First, we conducted the nerve explants culture to determine the potential role of omeprazole in rescuing nerve degeneration in wild-type and AhR-knockout mice. Second, both wild-type and AhR-knockout mice were subjected to chronic constrictive nerve injury and ten were treated with AhR agonist (omeprazole). These animals were finally examined in terms of neurobehavior (Von Frey, Hot plate, Catwalk gait analysis) and electrophysiology. Animal tissues analyzed with immunohistochemistry included the paw skin, crush nerve, dorsal root ganglion, dorsal spinal cord, and cerebral cortex or hippocampus.

## 2. Results

### 2.1. Modulation of AhR Expression Related to Nerve Degeneration and Regeneration

**Figure 1:**
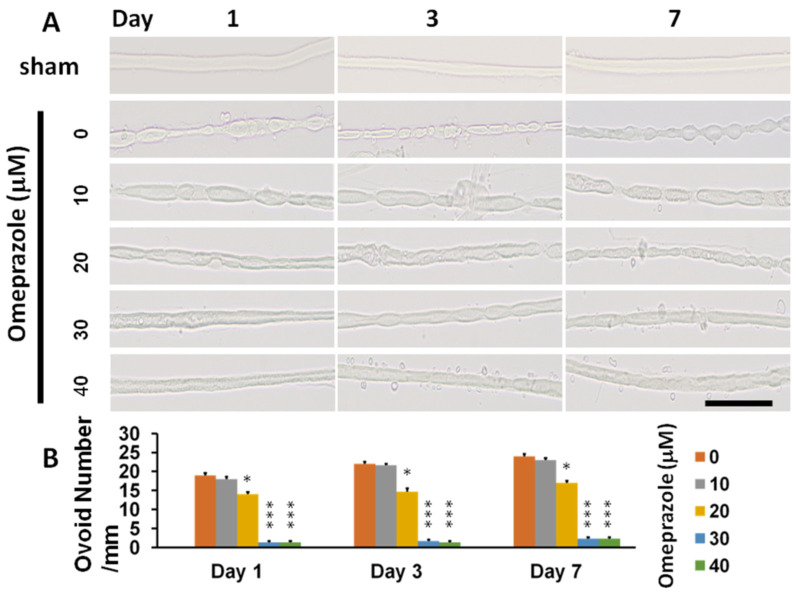
Assessment of AhR agonist (omeprazole) in preventing nerve degeneration in the nerve explant culture. The main trunk of the sciatic nerve was harvested from C57BL/6J mice with an age of one week and resected to 3 mm in length for culture. The nerve explant culture was subjected to different dosages of omeprazole and the ovoid numbers were calculated at the different time profiles to assess the protective effect. (**A**) The illustration of ovoid formation in different concentrations of omeprazole observed at different time points. (**B**) The quantitative analysis of ovoid number in the different treatment groups and time points. N = 3, Bar length = 100 μm; *: *p* < 0.05; ***: *p* < 0.001.

To assess nerve degeneration, the ovoid number in nerve explant culture is an essential method. Sciatic nerves from the C57BL/6J were resected into 3 mm-long segments for nerve explant culture. We found significantly increased ovoid numbers on day-1, 3 and 7 after CCI. When cultured with omeprazole (at concentrations of 0 to 40 μM), ovoid numbers showed dose-dependent decreases with a plateau at 30 μM, but no further increase in protection even at the maximum dose of 40 μM (Figure 1A,B). Omeprazole at 30 μM was taken as the optimal dosage for the remaining study. To assess the protective role of AhR in nerve degeneration, we conducted the nerve explant culture either from wild-type (C57BL/6J), AhR-knockout (AhR−/−) animals, or those intervened by AhR agonist omeprazole of 30 μM, and the results were assessed on day-3.

**Figure 2:**
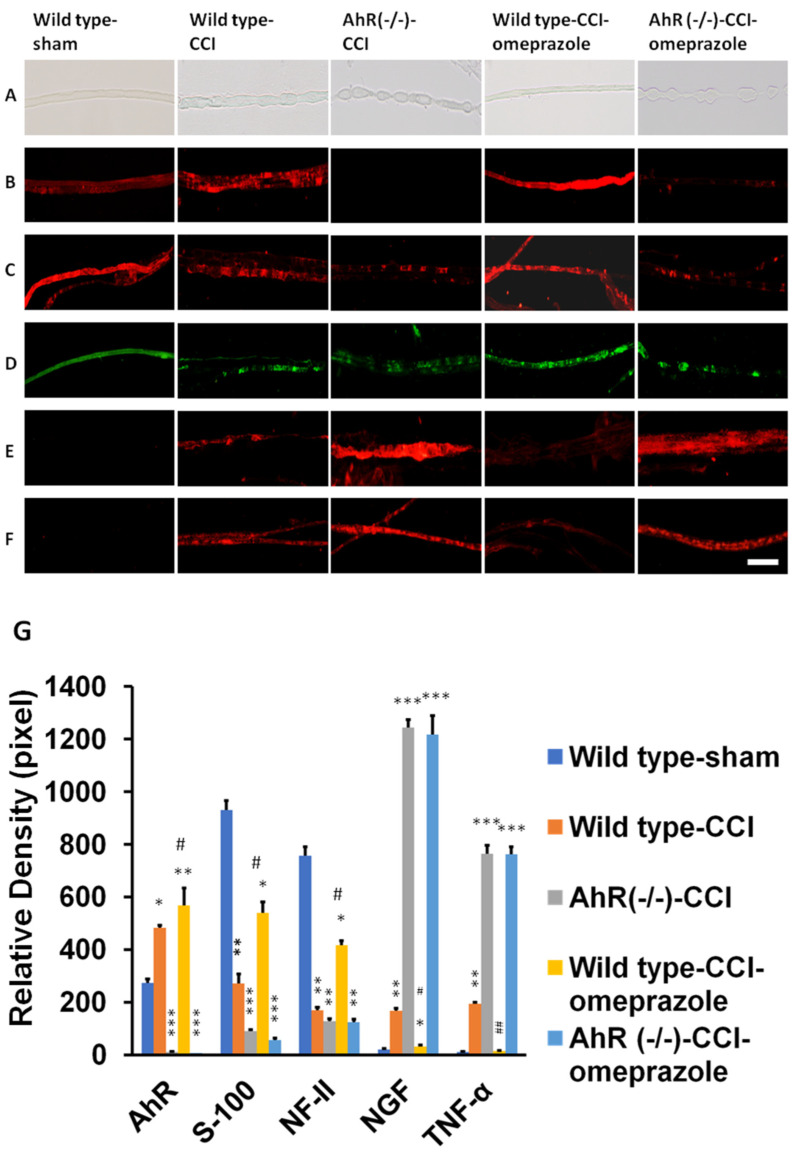
Modulation of AhR either by deletion or AhR agonist (omeprazole) in the nerve explant culture. Nerve explant either from wild or AhR-deleted mice was harvested and subjected to different treatment profiles observed on day-3. (**A**) Representatives of ovoid number in a light microscope related to different treatments. (**B**) Representatives of AhR in different treatments. (**C**) The expression of S-100 in different treatment groups. (**D**) The expression of NF-II in the different treatment groups. (**E**) The expression of NGF in different treatment groups. (**F**) The expression of TNF-α in different treatment groups. (**G**) The quantitative analysis of the target proteins in the different treatment groups. N = 3, Bar length = 100 μm, omeprazole concentration: 30 μM, *: *p* < 0.05; **: *p* < 0.01; ***: *p* < 0.001 indicated the value related to the group of Wild type-CCI. Wild type-sham; Wild type-CCI; AhR (−/−)-CCI; Wild type-CCI-omeprazole; AhR (−/−)-CCI-omeprazole: see text. # and ## indicated the value of wild type-CCI-omeprazole realtive to wild type-CCI.

Figure 2A shows increased ovoid numbers in wild-type animals, and further increments in AhR−/− animals, but such changes were attenuated with omeprazole treatment. The omeprazole had no effect in protection in AhR−/− animals. Figure 2B shows the increased expression of AhR in nerve degeneration (wild type) compared with sham. There was only a subtle expression of AhR in Ah−/− and a mild increase in AhR in wild-type animals treated with omeprazole. There was no definite increase in AhR expression in AhR−/− animals treated with omeprazole. To assess AhR in modulating nerve degeneration based on immunohistochemistry, we found significantly fewer nerve-regeneration-associated proteins (such as S-100 and neurofilament) in wild-type animals, and a further escalated drop in animals with AhR knockout, but such changes were attenuated by omeprazole. We also observed no definite improvement of S-100 and neurofilament in groups of AhR−/− treated with omeprazole (Figure 2C,D). Figure 2E,F shows the reciprocal expressions in NGF and TNF-α in the wild-type animals, and further escalation in AhR−/− animals, and such changes were alleviated by omeprazole. Again, there was no protection effect of omeprazole on AhR−/− animals in NGF and TNF-α expression.

### 2.2. Omeprazole Interacts with the Molecular Surface Representation of the AhR Binding Site (Ser36)

**Figure 3:**
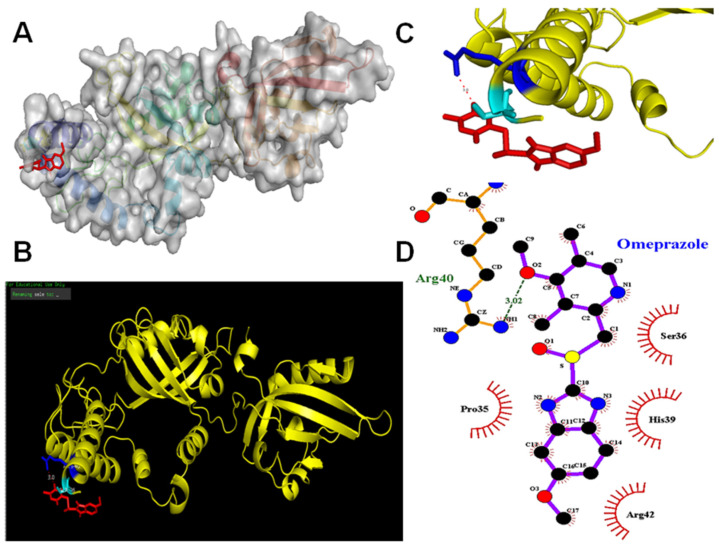
Omeprazole activated the AhR (Ser36) site by a molecular docking simulation. The chemical structure of omeprazole revealed binding modes in the AhR (Ser36) site by virtual screening. General and local overviews of the best interaction after automated docking of binding omeprazole (red) to the active site of AhR are shown. (**A**) Surface representation of the ligand–receptor binding. Omeprazole (red) and AhR are shown in surface representation. (**B**) Local interaction positions of the AhR (Ser36) active site (blue). The cave entrance of the zoom-in binding mode of AhR and omeprazole (red) is shown. (**C**) The molecular structure of human AhR with Ser 36 is depicted as a ribbon diagram, with helices and loops. A molecular surface representation of the binding mode of AhR and omeprazole is shown targeting Ser36 representation. The 3D representation of the interactions of omeprazole binding to the AhR active site was generated by PyMOL. (**D**) LIGPLOT generates schematic diagrams of 2D ligand–protein interaction diagrams. The interactions shown are those mediated by hydrogen bonds and by hydrophobic contacts.

We used ligand–protein-docking simulations to study molecular recognition. Molecular docking modeling uses PyMOL, which is an in silico analysis employed to determine possible binding of macromolecules or small compounds in their interaction with a receptor, thereby revealing their molecular interplay. Based on the above approach, results from animating 3D structures indicate omeprazole is a direct target of the AhR binding site (Ser36) site (Figure 3A–C). LIGPLOT further generates a schematic 2-D representation of protein–ligand complexes, supporting omeprazole’s direct interaction with AhR at site Ser36 (Figure 3D).

### 2.3. Knockout of AhR Aggravates Neurobehavior and Effects Attenuated by AhR Agonist

**Figure 4:**
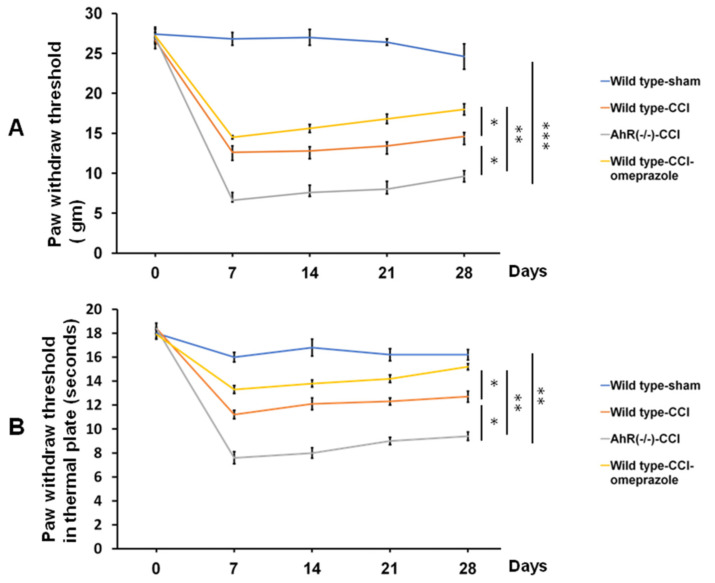
The assessment of neurobehavior by Von Frey and hot plate tests in CCI allocated to different treatment groups related to different time points. (**A**) Quantitative analysis of the paw withdrawal threshold by a Von Frey test in the different treatment groups related to different time points. (**B**) Quantitative analysis of paw thermal withdrawal threshold by hot plate tests in the different treatment groups related to different time points. N = 6, Wild type-sham; Wild type-CCI; AhR (−/−)-CCI; Wild type-CCI-omeprazole: see text. *: *p* < 0.05; **: *p* < 0.01; ***: *p* < 0.001.

For the assessment of neuropathic pain, mechanic or cold allodynia and thermal hyperalgesia were generally used [20,21,27]. In animal models of neuropathic pain, both the Von Frey and hot plate tests are used to assess mechanical allodynia and thermal hyperalgesia, respectively, based on our previous investigation. In animal models of neuropathic pain, both the Von Frey and hot plate tests are used to assess mechanical allodynia and thermal hyperalgesia, respectively. Chronic constriction injury caused an elevated and persistent mechanical allodynia and thermal sensitivity. The lowered threshold to mechanical allodynia in wild-type animals 7 days after CCI persisted for >28 days. In the AhR (−/−) group, compared with the wild-type group, further threshold lowering of mechanical allodynia was noted on day-7, with this trend compared with the wild type, persisting up to 28 days following nerve injury. The deterioration of a higher pain threshold was attenuated by omeprazole treatment (Figure 4A). The lowered withdrawal threshold for thermal sensitivity appeared in wild-type animals 7 days after the surgery, persisting for 28 days. The effect on thermal sensitivity threshold was further reduced on day-7 in the AhR (−/−) animals, when compared with the wild-type animals, and such an effect progressively increased towards day-28. Omeprazole treatment attenuated this response (Figure 4B). AhR knockout therefore predisposed the development of neuropathic pain, and such an effect was attenuated by the AhR agonist. 

The Catwalk gait analysis is a useful measurement of altered gait after CCI [21]. Our results of the Catwalk test are shown in Table 1. After CCI, we found shortened standing time (stand phase in msec), and deletion of AhR further reduced standing time, while omeprazole attenuated the effects. In the swing phase analysis, we found the opposite results. The data of printed area, single stance, and maximum contact maximum intensity showed the same trends as in the standing phase.

The muscle weight is an indirect index of nerve degeneration. After CCI, muscle weights in wild-type animals (wild type-CCI) significantly dropped (0.082 ± 0.003 gm) as compared with sham animals (wild type-sham) (0.16 ± 0.006 gm) (*p* < 0.001). In AhR−/− animals (AhR (−/−)-CCI), their muscle weights dropped further to 0.057 ± 0.003 gm (*p* < 0.001), and this change was attenuated by omeprazole (wild type-CCI-omeprazole) (0.11 ± 0.003 gm) (*p* < 0.001).

### 2.4. Histomorphology of Crushed Nerves from CCI and in AhR-Knockout Animals and the Attenuation Effect by AhR Agonist

**Figure 5:**
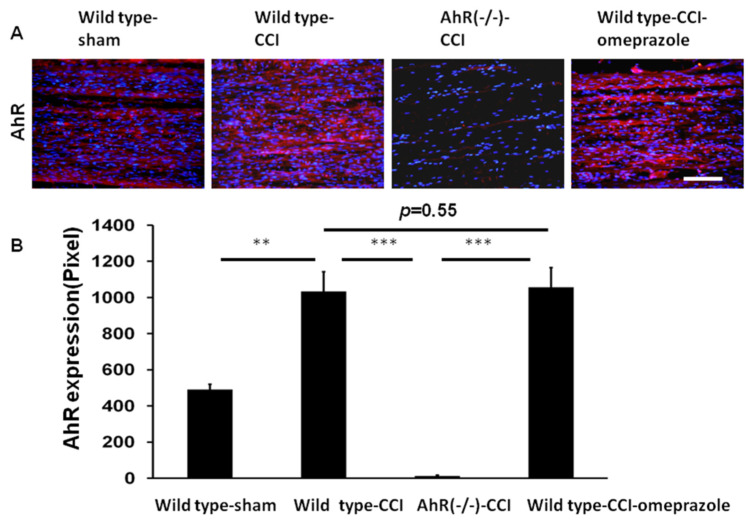
AhR expressions merged with DAPI at the distal end of the nerve 28 days after CCI. (**A**) Expression of AhR in distal end of nerve measured by pixel in the different treatment groups (**B**) The quantitative analysis of AhR expression by pixel in different treatment groups. N = 6, Bar length = 100 μm. **: *p* < 0.01; ***: *p* < 0.001; Wild type-sham; Wild type-CCI; AhR (−/−)-CCI; Wild type-CCI-omeprazole: see text. Red: AhR; blue: DAPI.

In studies on AhR expression after traumatic brain injury or stroke, elevated expressions of AhR occur in the injured nervous system [16,17,18]. In the analysis of nerve tissues obtained after CCI, wild type-CCI animals showed a marked increase in AhR expression compared with wild type-sham. The AhR (−/−)-CCI animals showed a minimal expression in AhR, and this level change was attenuated by omeprazole (wild-CCI-omeprazole) (Figure 5A,B).

**Figure 6:**
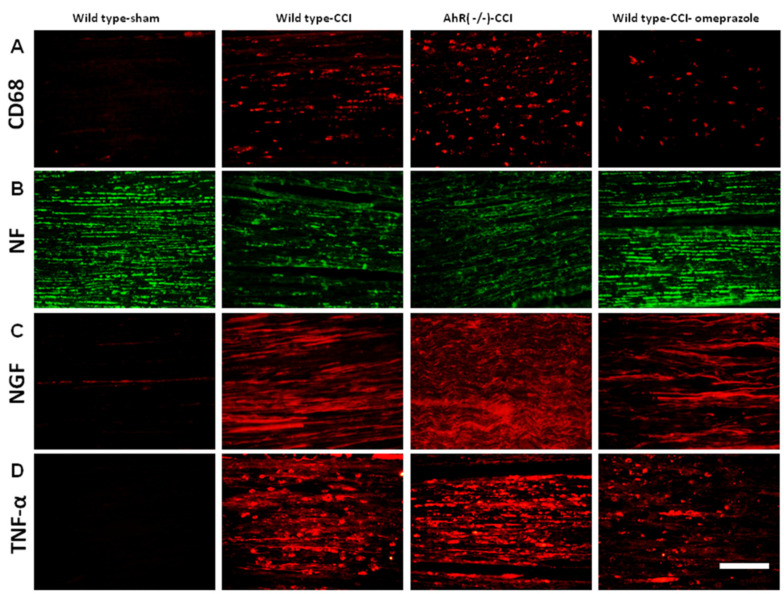
The expression of nerve degeneration and the associated inflammatory proteins in animals subjected to CCI 28 days after injury. (**A**) The expression of CD 68 in the distal end of nerve measured in different treatment groups. (**B**) The expression of neurofilament in different treatment groups. (**C**) The expression of NGF in different treatment groups. (**D**) The expression of TNF-α in different treatment groups. N = 6, Bar length = 100 μm; Wild type-sham; Wild type-CCI; AhR (−/−)-CCI; Wild type-CCI-omeprazole: see text.

In our previous study, we found higher expressions of CD 68, NGF, and TNF-α with a reciprocal decrease in neurofilament, and these findings suggested nerve degeneration [20,21]. We harvested distal ends of nerve for analysis, and we found higher expressions of CD 68, NGF, and TNF-α following injury in wild-type animals and further elevation in AhR−/− animals, while omeprazole attenuated such changes. However, the expression of neurofilament showed the opposite trends (Figure 6A–D). Statistical results are detailed in Table 2. 

### 2.5. Increased Pain Threshold in the Somatosensory System after CCI and in Ahr-Knockout Animals and the Attenuation Effect by AhR Agonist

**Figure 7:**
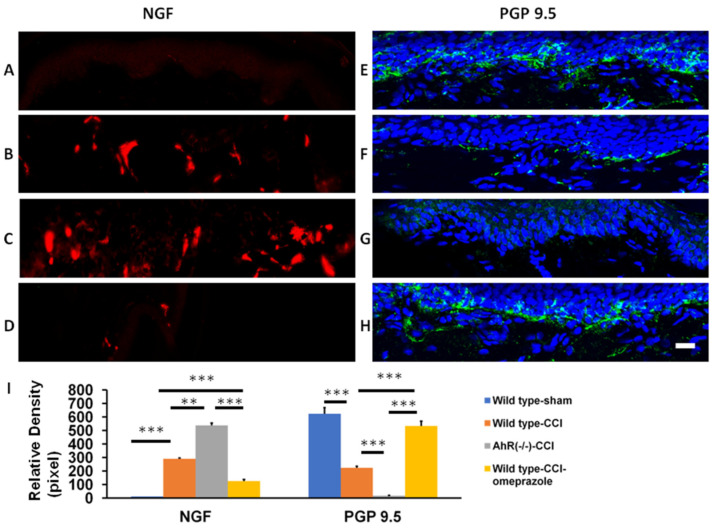
Assessment of the alteration of inflammation and regeneration in paw skin after CCI by modulation of AhR. Either wild or AhR-deleted mouse received CCI injuries and then were subjected to omeprazole treatment. (**A**) The expression of NGF in the sham group. (**B**) The expression of NGF in the wild group. (**C**) The expression of NGF in the AhR−/− group. (**D**) The expression of NGF in the wild+ omeprazole group. (**E**) The expression of PGP 9.5 in the sham group. (**F**) The expression of PGP 9.5 in the wild group. (**G**) The expression of PGP 9.5 in the AhR−/− group. (**H**) The expression of PGP 9.5 in the wild+ omeprazole group. (**I**) The quantitative analysis measured by pixel in the different treatment groups. Blue: DAPI; N = 6; Wild type-sham; Wild type-CCI; AhR (−/−)-CCI; Wild type-CCI-omeprazole: see text; bar length-100 μm; **: *p* < 0.01; ***: *p* < 0.001.

CCI induced neuropathic pain with the characteristic of altered histomorphology in structures from the paw skin to the somatosensory cortex [20,21,22]. No NGF expression appeared at the paw skin in the sham group. CCI induced a significant increase in the wild-type group. Further escalation was found in the AhR−/− group. Such a rise in NGF level in the wild-type group was not found in the omeprazole group (Figure 7A–D). In contrast, in the sham group, PGP 9.5 appeared extensively at the epidermal–dermal junction. CCI induced a marked reduction in PGP 9.5 expression in the wild-type group and a further reduction in the AhR−/− group. PGP 9.5 expression was restored in the omeprazole group (Figure 7E–I). 

**Figure 8:**
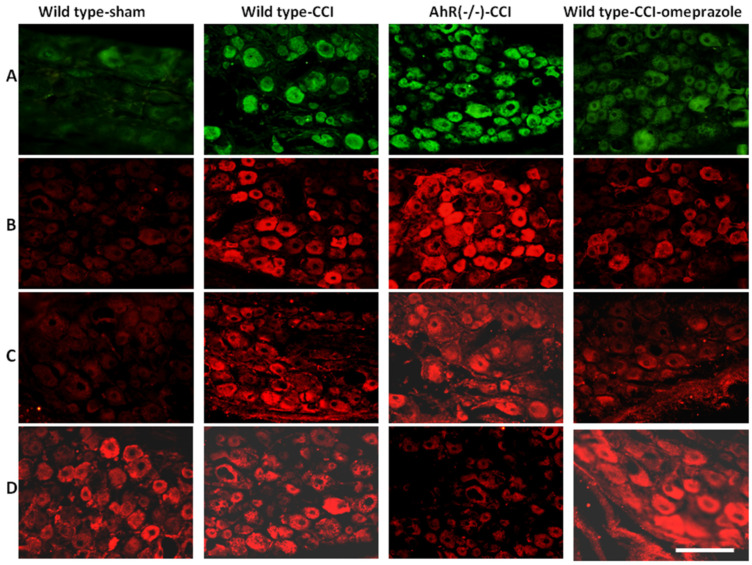
The alteration of the inflammatory response and AhR over the spinal dorsal root ganglion in a CCI model modulated by AhR. Either wild or AhR-deleted mouse received CCI injuries and then were subjected to omeprazole treatment. The dorsal root ganglion was obtained for the analysis 28 days after CCI. (**A**) Expression of NGF in the different treatment groups. (**B**) Expression of TNF-α in the different treatment groups. (**C**) Expression of synaptophysin in the different treatment groups. (**D**) Expression of AhR in the different treatment groups. Bar length = 100 μm; N = 6; Wild type-sham; Wild type-CCI; AhR (−/−)-CCI; Wild type-CCI-omeprazole: see text.

The dorsal root ganglion is a relay center in the development of neuropathic pain as measured by the expression of NGF, TNF-α, and synaptophysin. These proteins were minimally expressed in the sham group. Significant expressions of NGF, TNF-α and synaptophysin at the dorsal root ganglion were found in the wild-type group (Figure 8A–C). Wild type-CCI animals showed a marked increase in AhR expression compared with wild type-sham. The AhR (−/−)-CCI animals showed a minimal expression in AhR, and this level change was attenuated by omeprazole (wild-CCI-omeprazole) (Figure 8D). Detailed statistical results are shown in Table 3. In summary, AhR deletion sped up and escalated these inflammation-associated protein expressions, while AhR agonist alleviated this response.

**Figure 9:**
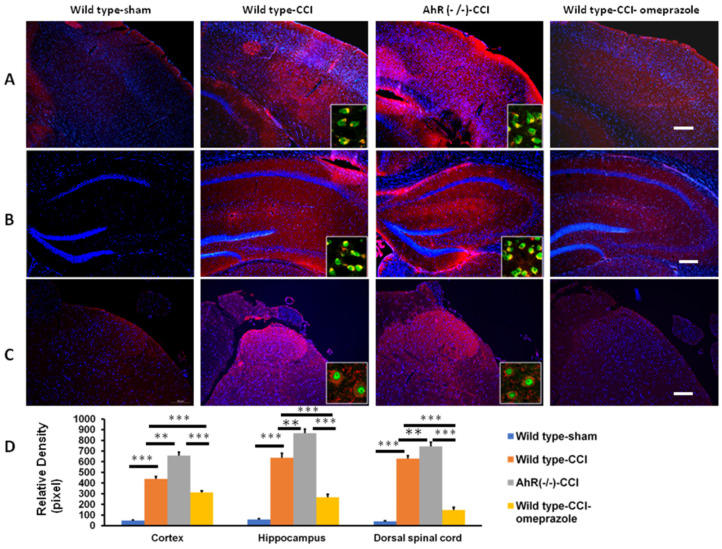
The expression of inflammatory cytokines in the central nervous system in CCI subjected to the modulation of AhR. Either wild or AhR-deleted mouse received CCI injuries and then were subjected to omeprazole treatment. The somatosensory cortex, hippocampus, and dorsal spinal cord were obtained for analysis 28 days after injury (**A**) Expression of TNF-α in the somatosensory cortex in the different experimental groups. (**B**) Expression of TNF-α in the hippocampus in the different experimental groups. (**C**) Expression of TNF-α in the dorsal spinal cord in the different experimental groups. (**D**) Quantitative analysis of TNF-α in different groups. Magnification box indicates the imaging fusion of TNF-α and Neu-N (green). DAPI = blue; Bar length = 100 μm; N = 6; **: *p* < 0.01; ***: *p* < 0.01. Wild type-sham; Wild type-CCI; AhR (−/−)-CCI; Wild type-CCI-omeprazole: see text.

TNF-α expression over the somatosensory cortex, hippocampus, and dorsal spinal cord was the typical presentation in response to CCI. The sham group showed only minimal expressions of TNF-α over the somatosensory cortex, hippocampus, and dorsal spinal cord. A high expression of TNF-α was noted in the wild-type group. Further elevated expression was found in the AhR−/− group. Such changes were attenuated by omeprazole treatment (Figure 9A–D).

**Figure 10:**
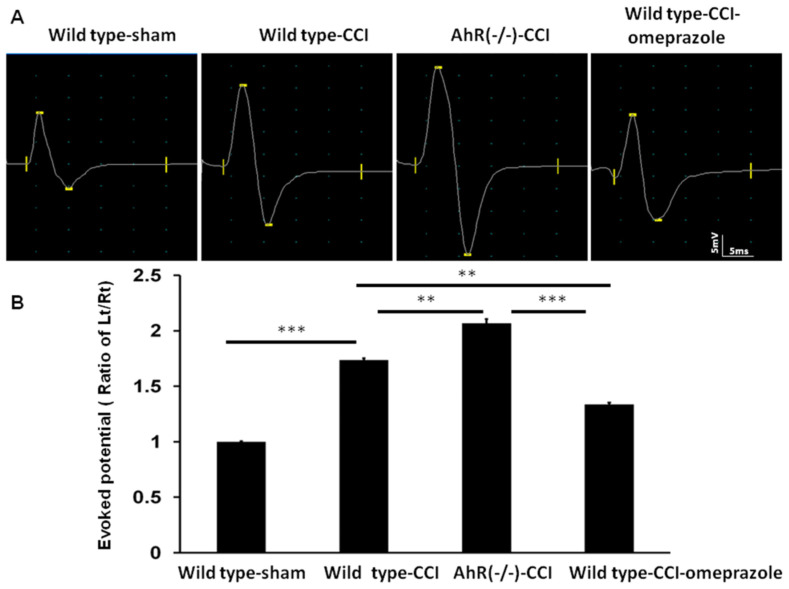
Alteration of somatosensory evoked potential in CCI in the different treatment groups. Either wild or AhR-deleted mouse received CCI injuries and then were subjected to omeprazole treatment. The somatosensory evoked potential was measured 28 days after CCI. (**A**) A representative of somatosensory evoked potential in the different treatment groups. (**B**) The quantitative analysis of the amplitude of evoked potential in the different groups. N = 6; Wild type-sham; Wild type-CCI; AhR (−/−)-CCI; Wild type-CCI-omeprazole: see text. **: *p* < 0.01; ***: *p* < 0.001.

In our previous study, we found that an increase in somatosensory evoked potential is highly correlated with the severity of neuropathic pain [21]. Our current study found that, after CCI, evoked potential increased in amplitude in the wild-type group. Further increase was noted in AhR (−/−) animals and such changes were attenuated by omeprazole treatment. The amplitude in the wild-type group was >1.7-fold larger compared with the sham group (*p* < 0.01). The AhR−/− animals showed a further amplitude increase to 2.1-fold (*p* < 0.001). Omeprazole treatment lowered it to 1.3-fold (*p* < 0.001) (Figure 10A,B). These findings indicated that the CCI of the peripheral nerve caused significant alterations in the electrophysiology of the sensorimotor cortex, and AhR knockout further aggravated the change, while omeprazole attenuated this response. 

## 3. Discussion

Our principal finding is that the modulation of AhR appeared to play a significant role for the development in neuropathic pain. The deletion of AhR significantly contributed to the occurrence of neuropathic pain while AhR agonist counteracted such an effect. Significant expression of AhR in the injured nerve was observed in the wild-type animals, either in the in vitro or in vivo study. AhR agonist (omeprazole) exerted a protection effect by attenuating the associated inflammatory response. In this study, we first used both the nerve explant culture and AhR-knockout mice to assess the role of AhR in the modulation of neuropathic pain. Our results justify the application of AhR agonist (omeprazole) in the treatment of neuropathic pain in the future.

In our previous investigation, we found that AhR regulated the energy balance, insulin sensitivity, and glucose metabolism in the diabetes condition involved in the regulation of the VEGFβ- and TGFα pathway [28,29]. The modulation of VEGF-β and TGF-α by AhR also contributed to the development of nerve injury and neuropathic pain [30,31]. The expression of the AhR has been studied in several animal models based on immunohistochemistry and hybridization in situ [7]. Specific temporal and spatial patterns of the AhR messenger RNA (mRNA) expression were reported in the mouse cortex, hippocampus, cerebellum, olfactory bulb, and rostral migratory stream [6,8,9]. The regulation of the AhR expression in the nervous system depends not only on internal stimuli, but also on external stimulation due to traumatic brain injury (TBI) or stroke [18]. AhR provides a potential therapeutic target for treating depression induced by chronic pain. Modulation of AhR signaling could be a valuable approach to alleviate the comorbidity of chronic pain and depression [32]. Chronic constrictive nerve injury is known to cause significant alteration in inflammatory response from the skin to the spinal cord, as well as the somatosensory cortex [20,21,22]. In this study, nerve injury caused significant elevation of AhR expression in the injured nerve, implicating a protective effect on nerve injury. Deleting AhR in vitro or in vivo caused further nerve degeneration. The external AhR ligand (omeprazole) exerted a protective effect. AhR expression took place in response to nerve injury, and it is essential for nerve protection.

Omeprazole (Prilosec) is a benzimidazole derivative and an AhR activator and ligand [24,25,26]. It is known to have anti-inflammatory effects in models of lung injury and neuropathic pain [7,26,33]. AhR agonists, either omeprazole or statin, prevent the release of proinflammatory cytokines, and maintain the structural integrity of the sciatic nerve from CCI-induced or crush nerve injury [33,34]. However, no investigation has yet been reported on how this involves AhR. In this study, the docking experiment found that omeprazole interacted with the AhR site (Ser36). In the nerve explant culture or CCI model, AhR deletion aggravated nerve degeneration, while AhR restored the nerve damage. Our results further confirmed that AhR plays a crucial role in the modulation of nerve injury. 

It is now recognized that NGF plays a key role in nociception by sensitizing nociceptors in the peripheral nervous system [35,36]. The expression of NGF in the paw skin is highly correlated to the severity of nerve injury [21]. The administering of NGF antiserum at the site of injury alleviates the onset of hyperalgesia [37]. Reduced expression of PGP 9.5 in axons of the epidermis and dermis is associated with neuropathic pain [35], and the restoration of PGP 9.5 fibers reflects the recovery of neuropathic pain [20]. In this study, an elevated expression of NGF was found in the wild-type animals, and it further increased after AhR deletion, but it was restored by administering AhR agonist. On the contrary, PGP 9.5 was reduced in wild-type animals, and it was further lowered after AhR deletion, but it was recovered by the AhR agonist. It further confirmed the hypothesis that AhR contributes to the development of neuropathic pain. 

Dorsal root ganglion cells are located in the intervertebral foramen of the spinal cord and involve sensory neurons. These cells respond to peripheral nerve injury. The increased expressions of TNF-α, NGF, and synaptophysin we found reflected the intensity of the neuropathic pain in either the in vitro or in vivo study. Attenuations of these inflammatory cytokines parallel reduced neuropathic pain [20,21,22,38]. In this study, we found that CCI induced significant expressions of TNF-α, NGF, and synaptophysin at the dorsal ganglion cells, and deletion of AhR further aggravated the inflammatory response. Administering the AhR agonist attenuated the inflammatory effects. These data of inflammatory response at the spinal dorsal root ganglion intervened by AhR deletion or AhR ligands supported the hypothesis that AhR plays a crucial role in regulating neuropathic pain.

Persistent chronic pain not only leads to sensory dysfunction, but also various brain disorders, involving cortical or subcortical dysfunction [39]. The physiology and structural remodeling of the learning circulatory of the hippocampus are reported with stress or chronic pain [40]. In our previous studies, we reported that the severity of chronic constriction injury is highly correlated with the intensity of the inflammatory response at the hippocampus. Additionally, attenuation of the neuropathic pain is associated with a lowered expression of the inflammatory response at the hippocampus [8,21,22,38]. In addition, altered evoked potentials at the cerebral cortex and dorsal spinal cord reflect neural plasticity and response to nerve damages [41]. In our previous study, the graded amplitude increases of evoked potential were highly correlated with the severity of nerve damage [21]. The attenuation of the injury is associated with reduced evoked potential [20,22,38]. In this study, high expressions of TNF-α from the dorsal spinal cord and hippocampus to the somatosensory cortex were demonstrated in the wild-type animals, but they were further escalated after AhR deletion. These effects were attenuated by omeprazole treatment. In addition, the changes in evoked potential at the somatosensory cortex we found are in line with the severity of neuropathic pain and related to both behavior and biochemistry [21]. Both electrophysiological and molecular biology data are in support of the fact that AhR is critically involved in the regulation of neuropathic pain.

The initiation and maintenance of neuropathic pain are mediated through the neuronal intrinsic pathways as well as the peripheral immune system [19]. After tissue injury, inflammation is generated by activating innate immune cells and glial cells. The immune-active substances, including cytokines, neurotrophic factors, and chemokines, as released from the immune cells, generate local actions leading to a more serious generalized immune response and sensory nerve sensitization [42,43]. A key feature of the microglial response to nerve trauma is microgliosis, which involves microglial changes in morphology and proliferation. Following peripheral nerve injury, apart from morphological changes, spinal microglia proliferate within 2 to 3 days, reaching a maximal level in 4 to 7 days [44].Normally, microgliosis after nerve injury is a brief and self-limited event, and microglia return to the normal level within weeks. Microgliosis induced by nerve injury occurs concomitantly with the development of pain hypersensitivity. Accordingly, blocking microgliosis attenuates pain behaviors [45,46,47]. AhR is widely expressed in several brain cell types, mostly neurons, with some expression in astrocytes and microglial cells [6]. A limitation of this study was that we did not assess the spatial and temporal effects of microglia affected by AhR, which might contribute to pathological development of neuropathic pain. 

## 4. Materials and Methods

### 4.1. Sciatic Nerve Explants Culture from Mice

We obtained C57BL/6 mice (4–5 weeks old, 20–22 g) from the National Applied Research Laboratories (NAR labs, Taipei, Taiwan), and AhR-knockout mice (B6.129Ahrtm1Bra/J) (6–8 weeks old, 18–22 g) from the Jackson laboratory (Bar Harbor, ME, USA). The sciatic nerve was first harvested from both wild-type and AhR (−/−) C57BL/6J mice, and then 3 mm-thick nerve sections were cultured in calcium/magnesium-free Hank’s buffered solution (HBSS). Under a microscope, the nerve tissue was isolated from the surrounding connective tissues, and only single nerve bundles were analyzed. To determine the effects of AhR agonist, at doses on the explant nerves, omeprazole (doses from 0 to 40 μM) was added to the sciatic nerve cultures. The culture medium was DMEM with 10% FBS and 5% PS, at the condition of 5% CO_2_ and 37 °C for 1 to 7 days. Finally, nerve tissues were fixed in 4% paraformaldehyde for 6 h, and observed under a light microscope. The ovoid number in each 500 μm length of nerve was calculated for statistical analyses. The nerve explant cultures were categorized into Wild type-sham, Wild type-CCI, AhR (−/−)-CCI, Wild type-CCI-omeprazole, and AhR (−/−)-CCI-omeprazole. 

### 4.2. Animal Model

To create the CCI rat model, the animal was put under gas anesthesia (4% isoflurane induction, 1–2% isoflurane for maintenance). Each animal was given a single loose surgical ligation around the left sciatic nerve. The surgical wound was closed with 4-0 silk sutures in layers, and the animal allowed to recover. The 4 groups of animals were: Wild type-sham, Wild type-CCI, AhR (−/−)-CCI, and Wild type-CCI-omeprazole. Omeprazole (Cayman chemical, Ann Arbor, MI, USA) was given orally (50 mg/kg/day) one day after CCI, and continued until day 14 [33]. After surgery, food and water were provided ad libitum before and after experiments. Animals were kept in a temperature-controlled room (20 °C), and they were exposed to a controlled light/dark cycle of 12/12 h. All animals were treated and cared for in accordance with the guidelines of the Institutional Animal Care and Use Committee or Panel of Taichung Veterans General Hospital.

Animals received the following behavioral tests: mechanical allodynia, thermal hyperalgesia, and CatWalk XT system gait analysis 3 days before surgery to establish baseline measurements, and then on a weekly basis until the end of experiments. Thereafter, animals underwent evoked potential recordings each month following CCI. They were also used for studies 4 weeks after CCI, on histomorphology and inflammatory responses from the skin to the brain.

### 4.3. Mechanical Allodynia and Thermal Hyperalgesia

A technician blinded to the animal grouping evaluated the thermal hyperalgesia and mechanical allodynia. During the mechanical allodynia test, a mouse was placed on a customized platform fixed in a transparent acrylic chamber (dimensions: 20 × 20 × 20 cm^3^). The customized platform was 20 × 20 cm^2^ and made of 5 mm-thick acrylic plate. It contained 2 mm-diameter holes in a 5 mm grid of perpendicular rows covering the entire area of the platform. A test trial consisted of applying a single Von Frey hair (Touch-Test Sensory Evaluator, North Coast Medical, Inc., Morgan Hill, CA, USA) to the hind paw at 5 s intervals 5 times, with the hind paw placed appropriately on the platform. If the hind paw withdrawal did not occur during the repeated 5 applications of a hair, a hair of a larger caliber was applied. When the hind paw had been withdrawn from a particular hair for either 4 or 5 times out of the 5 applications, the hair pressure calibrated in grams was considered to be the withdrawal threshold force. Thermal hyperalgesia was tested with our previous method using the hot plate (TSE Systems). The paw withdrawal latency, defined as the time from the paw in contact with the 52 °C hot plate to its withdrawal, was recorded with a timer. To prevent tissue damage, the maximum test time was 20 s [20].

### 4.4. CatWalk Automated Quantitative Gait Analysis

We used the published method on CatWalk gait analysis [21]. In brief, the CatWalk XT system consisted of a high-speed digital camera (sampling rate: 100 frames/second). The captured digital images were stored in a computer through an Ethernet connection. 

The Illuminated Footprint enabled the detection of intensity differences (gray levels from 0 to 255, color-coded) across an animal’s 4 paws. The 3D footprint intensity tab plots, on a 3D chart, the print intensity of the 4 paws for each frame during which the paws were in contact with the underlying glass plate. The 3D chart was able to be rotated in any direction. Quantitative analysis of the data from the CatWalk XT system included the following parameters: step sequence distribution, regularity index, print area, duration of swing and stance phases, and maximum contact intensity (intensity at maximum contact of a paw).

### 4.5. Electrophysiological Study

One month after CCI injury, sensorimotor evoked potentials were studied one day before euthanizing the animals. In brief, under anesthesia, the cerebral cortex was exposed on both sides, and two recording electrodes were each placed on the dural surface overlying the somatosensory area bilaterally (3 mm lateral, 2 mm posterior to the bregma). At the sciatic nerve, one cm proximal to the injured site, an electrical stimulus (a 20 mA pulse) was delivered through an active stimulating electrode. The reference needle electrode was positioned 2 cm from the recording electrode. The conduction latency and evoked potential were recorded. The frequency of recording was 20 to 200 Hz. To adjust for the effect of anesthesia, we converted the data of conduction latency and evoked potential to a ratio of the injured side divided by the normal side [21].

### 4.6. Immunohistochemical Analyses

Animals were anesthetized and perfused first with phosphate-buffered saline, and then with a 4% paraformaldehyde fixative. We then resected the following: the brain (cerebral cortex and hippocampus), spinal cord dorsal horn, dorsal root ganglia (bilateral L-4 to L-6 dorsal root ganglia), sciatic nerves (middle of the crushed site), and foot skin (hindlimb paw skin). They were then placed in 4% paraformaldehyde for 4 h before submerging in 30% sucrose at 4 °C overnight. Samples were subsequently embedded in Tissue-Tek O.C.T. Compound (Sakura) and rapidly frozen.

Serial 8 um-thick sections of all samples (skin, sciatic nerve, dorsal root ganglion, dorsal spinal cord, and brain) were cut on a cryostat and mounted on Superfrost Plus slides (MenzelGlaser, Braunschweig, Germany). Sections were subjected to immunohistochemical examination after reactions with antibodies against the following: AhR (Gene Tex, 1:250 dilution, Alton Pkwy Irvine, CA, USA), Neu-N (Millipore, 1:500 dilution, Burlington, MA, USA), NGF (Chemicon, 1:300 dilution, Osaki, Japan), PGP 9.5 (Abam, 1:500 dilution, Istanbul, Turkey), Neurofilament (Millipore, 1:500 dilution), CD68 (Chemicon, 1:200 dilution), S100 protein (Neomarkers, 1:400 dilution, Portsmouth, NH, USA), synaptophysin (Abcam, 1:200 dilution), and TNF-α (Abcam, 1:300 dilution). These antibodies were designed to detect, respectively, the following: inflammatory cells, Schwann cells, small neurosecretory vesicles, and inflammatory cytokines. Immunoreactive signals were visualized using goat anti–mouse Immunoglobulin G (fluorescein isothiocyanate, Jackson; 1:200 dilution) and anti–mouse Immunoglobulin G (rhodamine, Jackson; 1:200 dilution). Six tissue specimens in each group were cut into 8 mm-thick sections and stained with the antibody. All the fluorescent imaging was performed with the same laser power and exposure time by the Olympus BX40 Research Microscope (Hicksville, NY, USA). The IHC images were quantitatively analyzed by Image J and UN-SCAN-IT gelTM (Gel & Graph Digitizing Software Version 6.1, Provo, UT, USA). Areas occupied by the stained tissues were highlighted and measured for each section of sciatic nerve (n = 6 per group) and expressed in terms of the density (pixel) of all the resected tissue [20,21].

### 4.7. Molecular Docking Modeling

PyMOL is a widely popular macromolecular visualization system that uses the OpenGL Extension Wrangler Library (GLEW) and Free OpenGL Utility Toolkit (FreeGLUT). PyMOL uses a cross-platform widget toolkit (Tk) for the GUI widgets and can produce high-quality movies and images of macromolecules in different representations such as ribbon, cartoon, dot, surface, sphere, stick, and line. PyMOL can also extend to the protein–ligand modeling, molecular simulations (MS), and virtual screening (VS) unities in PyMOL. The computational drug discovery function of PyMOL has been successfully applied to find new drug candidates for various targets [48].

### 4.8. Statistical Analyses

Data were presented as mean ± standard error. Student’s *t*-test and repeated-measures ANOVA were used to compare inter-group differences. Statistical analyses were conducted using SPSS software (version 12, Chicago, IL, USA). Statistical significance was set at *p* < 0.05.

## 5. Conclusions

High expressions of AhR occurred in response to chronic constriction nerve injury, and AhR deletion aggravated the effect. The AhR agonist omeprazole alleviated such changes. We speculate that AhR agonist is a potential candidate for treating neuropathic pain.

## 6. Patents

There are patents resulting from the work reported in this manuscript.

## Figures and Tables

**Table 1:** Outcomes of various Catwalk gait analysis parameters at different time points in the various treatment groups.

		Day					*p* Value
		0	7	14	21	28	
Stands (msec)	Wild type-sham	245.2 ± 5.1	248.8 ± 6.1	253.5 ± 8.7	257.7 ± 3.9	255.2 ± 6.6	<0.001
	Wild type-CCI	244.2 ± 8.4	141.3 ± 11.1	162.2 ± 9.1	178.5 ± 11.5	186.8 ± 6.9	
	AhR(−/−)-CCI	244.7 ± 12.1	119.2 ± 5.4	125.7 ± 6.2	135.8 ± 6.4	142.2 ± 7.2	
	Wild type-CCI- omeprazole	246.2 ± 6.1	164.5 ± 8.4	183.1 ± 8.7	195.2 ± 3.9	216.8 ± 3.6	
Swings (msec)	Wild type-sham	115.8 ± 4.8	118.1 ± 3.1	117.8 ± 4.5	117.8 ± 3.3	116.5 ± 3.2	<0.001
	Wild type-CCI	114.2 ± 5.4	210.5 ± 2.1	192.7 ± 5.1	180.3 ± 9.3,	157.8 ± 2.9	
	AhR(−/−)-CCI	112.2 ± 4.8	319.8 ± 8.9	282 ± 8.1	238.7 ± 12.1	210.1 ± 9.7	
	Wild type-CCI omeprazole	115.1 ± 6.1	184 ± 6.6	162.2 ± 8.7	150.8 ± 9.9	135.3 ± 5.1	
Max Contact Max Intensity (Green Intensity)	Wild type-sham	235.2 ± 5.4	238.8 ± 8.4	237.8 ± 2.8	238.5 ± 1.9	236.8 ± 3.5	<0.001
	Wild type-CCI	235 ± 3.1	188.5 ± 3.2	197.8 ± 3.3	240.5 ± 4.2	214.7 ± 2.1	
	AhR(−/−)-CCI	236.2 ± 5.4	175.8 ± 6.2	178.8 ± 4.2	187 ± 2.4	195.3 ± 2.9	
	Wild type-CCI- omeprazole	237.3 ± 3.8	198.2 ± 3.4	210.5 ± 1.2	218.8 ± 3.3	228 ± 3.2	
Printed area (cm^2^)	Wild type-sham	0.315 ± 0.09	0.317 ± 0.08	0.318 ± 0.03	0.32 ± 0.03	0.319 ± 0.02	<0.001
	Wild type-CCI	0.319 ± 0.07	0.078 ± 0.01	0.138 ± 0.02	0.186 ± 0.02	0.247 ± 0.06	
	AhR(−/−)-CCI	0.318 ± 0.08	0.051 ± 0.002	0.094 ± 0.03	0.152 ± 0.03	0.194 ± 0.03	
	Wild type-CCI- omeprazole	0.320 ± 0.09	0.115 ± 0.01	0.180 ± 0.01	0.214 ± 0.04	0.282 ± 0.03	
Single Stance (msec)	Wild type-sham	125.2 ± 4.1	124.3 ± 3.6	123.8 ± 4.2	123.3 ± 2.9	126 ± 2.2	<0.001
	Wild type-CCI	125.8 ± 4.2	71 ± 2.9	78.3 ± 1.2	85.9 ± 2.8	91.8 ± 3.9	
	AhR(−/−)-CCI	126.7 ± 3.6	64 ± 1.8	67.8 ± 1.9	73.2 ± 2.2	79.5 ± 2.1	
	Wild type-CCI omeprazole	126.2 ± 6.3	83.5 ± 3.9	89.3 ± 3.3	93.3 ± 3.1	104.8 ± 5.5	

The data are presented as mean ± standard errors. Wild type-sham; Wild type-CCI; AhR (−/−)-CCI; Wild type-CCI-omeprazole: see text.

**Table 2:** Expression of regeneration and associated proteins in the distal end of neve.

	Wild Type-Sham	Wild Type-CCI	AhR(−/−)-CCI	Wild Type-CCI-Omeprazole	*p* Value
CD 68	3 ± 0.5	95.3 ± 3.3	143.7 ± 3.3	50.7 ± 2.9	*p* < 0.001
NF	981 ± 16.5	426 ± 13.1	287 ± 19.7	604 ± 57.4	*p* < 0.001
NGF	12.7 ± 1.2	399.3 ± 10.3	583 ± 16.6	180.3 ± 15.5	*p* < 0.001
TNF-α	7 ± 1.2	778 ± 22.2	830.3 ± 20.6	183 ± 16.5	*p* < 0.01

The data are presented as pixels and the method is depicted in the text. Wild type-sham; Wild type-CCI; AhR (−/−)-CCI; Wild type-CCI-omeprazole.

**Table 3:** Expression of the inflammatory proteins in the dorsal root ganglion.

	Wild Type-Sham	Wild Type-CCI	AhR(−/−)-CCI	Wild Type-CCI- Omeprazole	*p* Value
NGF	19 ± 1.2	244.3 ± 14.5	429.3 ± 12.4	147.7 ± 14.8	*p* < 0.001
TNF-α	9 ± 1.1	188.7 ± 5.5	477 ± 14.2	103.1 ± 4.1	*p* < 0.001
Synaptophysin	12.1 ± 0.6	155.2 ± 6.9	241.1 ± 4.9	89.2 ± 6.1	*p* < 0.001
AhR	102.7 ± 3.8	298.7 ± 6.4	14.1 ± 4.6	347.3 ± 14.5	*p* < 0.001

The data are presented as pixels and the method is depicted in the text.

## Data Availability

Not applicable.

## References

[1] Baron R., Tölle T.R. (2008). Assessment and diagnosis of neuropathic pain. Curr. Opin. Support. Palliat. Care.

[2] Treede R.D., Jensen T.S., Campbell J.N., Cruccu G., Dostrovsky J.O., Griffin J.W., Hansson P., Hughes R., Nurmikko T., Serra J. (2008). Neuropathic pain: Redefinition and a grading system for clinical and research purposes. Neurology.

[3] Sacerdote P., Niada S., Franchi S., Arrigoni E., Rossi A., Yenagi V., de Girolamo L., Panerai A.E., Brini A.T. (2013). Systemic administration of human adipose-derived stem cells reverts nociceptive hypersensitivity in an experimental model of neuropathy. Stem Cells Dev..

[4] Siniscalco D., Rossi F., Maione S. (2007). Molecular approaches for neuropathic pain treatment. Curr. Med. Chem..

[5] Guyot E., Chevallier A., Barouki R., Coumoul X. (2013). The AhR twist: Ligand-dependent AhR signaling and pharmaco-toxicological implications. Drug Discov. Today.

[6] Juricek L., Coumoul X. (2018). The Aryl Hydrocarbon Receptor and the Nervous System. Int. J. Mol. Sci..

[7] Jain S., Maltepe E., Lu M.M., Simon C., Bradfield C.A. (1998). Expression of ARNT, ARNT2, HIF1 alpha, HIF2 alpha and Ah receptor mRNAs in the developing mouse. Mech. Dev..

[8] Kimura E., Tohyama C. (2017). Embryonic and Postnatal Expression of Aryl Hydrocarbon Receptor mRNA in Mouse Brain. Front. Neuroanat..

[9] Moran T.B., Brannick K.E., Raetzman L.T. (2012). Aryl-hydrocarbon receptor activity modulates prolactin expression in the pituitary. Toxicol. Appl. Pharmacol..

[10] Filbrandt C.R., Wu Z., Zlokovic B., Opanashuk L., Gasiewicz T.A. (2004). Presence and functional activity of the aryl hydrocarbon receptor in isolated murine cerebral vascular endothelial cells and astrocytes. Neurotoxicology.

[11] Kubota A., Stegeman J.J., Woodin B.R., Iwanaga T., Harano R., Peterson R.E., Hiraga T., Teraoka H. (2011). Role of zebrafish cytochrome P450 CYP1C genes in the reduced mesencephalic vein blood flow caused by activation of AHR2. Toxicol. Appl. Pharmacol..

[12] Pravettoni A., Colciago A., Negri-Cesi P., Villa S., Celotti F. (2005). Ontogenetic development, sexual differentiation, and effects of Aroclor 1254 exposure on expression of the arylhydrocarbon receptor and of the arylhydrocarbon receptor nuclear translocator in the rat hypothalamus. Reprod. Toxicol..

[13] Li Y., Chen G., Zhao J., Nie X., Wan C., Liu J., Duan Z., Xu G. (2013). 2,3,7,8-Tetrachlorodibenzo-p-dioxin (TCDD) induces microglial nitric oxide production and subsequent rat primary cortical neuron apoptosis through p38/JNK MAPK pathway. Toxicology.

[14] Mukai M., Lin T.M., Peterson R.E., Cooke P.S., Tischkau S.A. (2008). Behavioral rhythmicity of mice lacking AhR and attenuation of light-induced phase shift by 2,3,7,8-tetrachlorodibenzo-p-dioxin. J. Biol. Rhythm..

[15] Petersen S.L., Curran M.A., Marconi S.A., Carpenter C.D., Lubbers L.S., McAbee M.D. (2000). Distribution of mRNAs encoding the arylhydrocarbon receptor, arylhydrocarbon receptor nuclear translocator, and arylhydrocarbon receptor nuclear translocator-2 in the rat brain and brainstem. J. Comp. Neurol..

[16] Cuartero M.I., Ballesteros I., de la Parra J., Harkin A.L., Abautret-Daly A., Sherwin E., Fernández-Salguero P., Corbí A.L., Lizasoain I., Moro M.A. (2014). L-kynurenine/aryl hydrocarbon receptor pathway mediates brain damage after experimental stroke. Circulation.

[17] Wójtowicz A.K., Szychowski K.A., Wnuk A., Kajta M. (2017). Dibutyl Phthalate (DBP)-Induced Apoptosis and Neurotoxicity are Mediated via the Aryl Hydrocarbon Receptor (AhR) but not by Estrogen Receptor Alpha (ERα), Estrogen Receptor Beta (ERβ), or Peroxisome Proliferator-Activated Receptor Gamma (PPARγ) in Mouse Cortical Neurons. Neurotoxic. Res..

[18] Xu K., Yang Z., Shi R., Luo C., Zhang Z. (2016). Expression of aryl hydrocarbon receptor in rat brain lesions following traumatic brain injury. Diagn. Pathol..

[19] Scholz J., Woolf C.J. (2007). The neuropathic pain triad: Neurons, immune cells and glia. Nat. Neurosci..

[20] Chiang C.Y., Liu S.A., Sheu M.L., Chen F.C., Chen C.J., Su H.L., Pan H.C. (2016). Feasibility of Human Amniotic Fluid Derived Stem Cells in Alleviation of Neuropathic Pain in Chronic Constrictive Injury Nerve Model. PLoS ONE.

[21] Chiang C.Y., Sheu M.L., Cheng F.C., Chen C.J., Su H.L., Sheehan J., Pan H.C. (2014). Comprehensive analysis of neurobehavior associated with histomorphological alterations in a chronic constrictive nerve injury model through use of the CatWalk XT system. J. Neurosurg..

[22] Sheu M.L., Chiang C.Y., Su H.L., Chen C.J., Sheehan J., Pan H.C. (2018). Intrathecal Injection of Dual Zipper Kinase shRNA Alleviating the Neuropathic Pain in a Chronic Constrictive Nerve Injury Model. Int. J. Mol. Sci..

[23] Jin U.H., Lee S.O., Safe S. (2012). Aryl hydrocarbon receptor (AHR)-active pharmaceuticals are selective AHR modulators in MDA-MB-468 and BT474 breast cancer cells. J. Pharmacol. Exp. Ther..

[24] Hu W., Sorrentino C., Denison M.S., Kolaja K., Fielden M.R. (2007). Induction of cyp1a1 is a nonspecific biomarker of aryl hydrocarbon receptor activation: Results of large scale screening of pharmaceuticals and toxicants in vivo and in vitro. Mol. Pharmacol..

[25] Shivanna B., Chu C., Welty S.E., Jiang W., Wang L., Couroucli X.I., Moorthy B. (2011). Omeprazole attenuates hyperoxic injury in H441 cells via the aryl hydrocarbon receptor. Free Radic. Biol. Med..

[26] Shivanna B., Jiang W., Wang L., Couroucli X.I., Moorthy B. (2011). Omeprazole attenuates hyperoxic lung injury in mice via aryl hydrocarbon receptor activation and is associated with increased expression of cytochrome P4501A enzymes. J. Pharmacol. Exp. Ther..

[27] Xing H., Chen M., Ling J., Tan W., Gu J.G. (2007). TRPM8 mechanism of cold allodynia after chronic nerve injury. J. Neurosci..

[28] Lai D.W., Lin K.H., Sheu W.H., Lee M.R., Chen C.Y., Lee W.J., Hung Y.W., Shen C.C., Chung T.J., Liu S.H. (2017). TPL2 (Therapeutic Targeting Tumor Progression Locus-2)/ATF4 (Activating Transcription Factor-4)/SDF1α (Chemokine Stromal Cell-Derived Factor-α) Axis Suppresses Diabetic Retinopathy. Circ. Res..

[29] Lee W.J., Liu S.H., Chiang C.K., Lin S.Y., Liang K.W., Chen C.H., Tien H.R., Chen P.H., Wu J.P., Tsai Y.C. (2016). Aryl Hydrocarbon Receptor Deficiency Attenuates Oxidative Stress-Related Mesangial Cell Activation and Macrophage Infiltration and Extracellular Matrix Accumulation in Diabetic Nephropathy. Antioxid. Redox Signal..

[30] Chen G., Zhang Y.Q., Qadri Y.J., Serhan C.N., Ji R.R. (2018). Microglia in Pain: Detrimental and Protective Roles in Pathogenesis and Resolution of Pain. Neuron.

[31] Wekerle H. (2018). Brain inflammatory cascade controlled by gut-derived molecules. Nature.

[32] Li S., Hua D., Wang Q., Yang L., Wang X., Luo A., Yang C. (2020). The Role of Bacteria and Its Derived Metabolites in Chronic Pain and Depression: Recent Findings and Research Progress. Int. J. Neuropsychopharmacol..

[33] Chanchal S.K., Mahajan U.B., Siddharth S., Reddy N., Goyal S.N., Patil P.H., Bommanahalli B.P., Kundu C.N., Patil C.R., Ojha S. (2016). In vivo and in vitro protective effects of omeprazole against neuropathic pain. Sci. Rep..

[34] Pan H.C., Yang D.Y., Ou Y.C., Ho S.P., Cheng F.C., Chen C.J. (2010). Neuroprotective effect of atorvastatin in an experimental model of nerve crush injury. Neurosurgery.

[35] Bennett D.L. (2001). Neurotrophic factors: Important regulators of nociceptive function. Neuroscientist.

[36] Koltzenburg M., Bennett D.L., Shelton D.L., McMahon S.B. (1999). Neutralization of endogenous NGF prevents the sensitization of nociceptors supplying inflamed skin. Eur. J. Neurosci..

[37] Herzberg U., Eliav E., Dorsey J.M., Gracely R.H., Kopin I.J. (1997). NGF involvement in pain induced by chronic constriction injury of the rat sciatic nerve. Neuroreport.

[38] Su H.L., Chiang C.Y., Lu Z.H., Cheng F.C., Chen C.J., Sheu M.L., Sheehan J., Pan H.C. (2018). Late administration of high-frequency electrical stimulation increases nerve regeneration without aggravating neuropathic pain in a nerve crush injury. BMC Neurosci..

[39] Zhao X.Y., Liu M.G., Yuan D.L., Wang Y., He Y., Wang D.D., Chen X.F., Zhang F.K., Li H., He X.S. (2009). Nociception-induced spatial and temporal plasticity of synaptic connection and function in the hippocampal formation of rats: A multi-electrode array recording. Mol. Pain.

[40] Liu M.G., Chen J. (2009). Roles of the hippocampal formation in pain information processing. Neurosci. Bull..

[41] Zelenka M., Schäfers M., Sommer C. (2005). Intraneural injection of interleukin-1beta and tumor necrosis factor-alpha into rat sciatic nerve at physiological doses induces signs of neuropathic pain. Pain.

[42] Siniscalco D., Giordano C., Rossi F., Maione S., de Novellis V. (2011). Role of neurotrophins in neuropathic pain. Curr. Neuropharmacol..

[43] Vallejo R., Tilley D.M., Vogel L., Benyamin R. (2010). The role of glia and the immune system in the development and maintenance of neuropathic pain. Pain Pract..

[44] Echeverry S., Shi X.Q., Zhang J. (2008). Characterization of cell proliferation in rat spinal cord following peripheral nerve injury and the relationship with neuropathic pain. Pain.

[45] Gu N., Peng J., Murugan M., Wang X., Eyo U.B., Sun D., Ren Y., DiCicco-Bloom E., Young W., Dong H. (2016). Spinal Microgliosis Due to Resident Microglial Proliferation Is Required for Pain Hypersensitivity after Peripheral Nerve Injury. Cell Rep..

[46] Guan Z., Kuhn J.A., Wang X., Colquitt B., Solorzano C., Vaman S., Guan A.K., Evans-Reinsch Z., Braz J., Devor M. (2016). Injured sensory neuron-derived CSF1 induces microglial proliferation and DAP12-dependent pain. Nat. Neurosci..

[47] Sorge R.E., Mapplebeck J.C., Rosen S., Beggs S., Taves S., Alexander J.K., Martin L.J., Austin J.S., Sotocinal S.G., Chen D. (2015). Different immune cells mediate mechanical pain hypersensitivity in male and female mice. Nat. Neurosci..

[48] Seeliger D., de Groot B.L. (2010). Ligand docking and binding site analysis with PyMOL and Autodock/Vina. J. Comput. Aided Mol. Des..

